# Hospital Security Searches Among Patients With Substance-Related Encounters

**DOI:** 10.1001/jamanetworkopen.2025.1068

**Published:** 2025-03-18

**Authors:** Sarah J. Nessen, Ashish Thakrar, Jeanmarie Perrone, Lin Xu, Rachel McFadden, Margaret Lowenstein

**Affiliations:** 1 Perelman School of Medicine, University of Pennsylvania, Penn Center for Emergency Care and Policy Research, Penn Center for Addiction Medicine and Policy, Philadelphia; 2 Division of General Internal Medicine, University of Pennsylvania Perelman School of Medicine, Philadelphia; 3 Department of Emergency Medicine, Penn Center for Addiction Medicine and Policy, University of Pennsylvania Perelman School of Medicine, Philadelphia; 4 Division of General Internal Medicine, Penn Center for Addiction Medicine and Policy, University of Pennsylvania Perelman School of Medicine, Philadelphia

## Abstract

**Importance:**

Hospital clinicians may request that security personnel search patients’ rooms and belongings in response to suspected possession or use of illicit substances. Although searches are intended to protect patient and employee safety, little is known about search outcomes or consequences for clinical care.

**Objective:**

To investigate the clinical context and outcomes of room searches among patients with substance-related hospital encounters.

**Design, Setting, and Participants:**

Retrospective cohort study of security incident reports and electronic health record data for patients with substance-related hospital encounters between July 2021 and July 2023 at an urban, academic hospital with approximately 1000 beds in Philadelphia, Pennsylvania. Participants were adults with *International Statistical Classification of Diseases and Related Health Problems, Tenth Revision* codes consistent with substance use disorders, chief concerns of drug overdose or withdrawal, and/or positive verbal screenings for opioid use who had emergency department visits or inpatient hospitalizations.

**Main Outcomes and Measures:**

Primary outcomes were search requests, items confiscated, and patient-directed discharges (PDDs). Secondary measures centered on the clinical context of searches, including location and receipt of medications for opioid use disorder.

**Results:**

There were 13 827 total substance-related hospital encounters over the study period involving 6985 unique patients (median [IQR] age, 47 [34-58] years; 3863 [55.3%] male; 3688 [52.7%] Black; 302 [4.3%] Hispanic; 2597 [37.2%] White). Over this time, security reported 576 room search requests, among which 457 (79.3%) were requests for searches among patients with substance-related hospital encounters. Nurses requested most searches (376 searches [82.3%]), and 195 searches (43.6%) resulted in no items confiscated. Confiscated items included syringes and unspecified paraphernalia (129 searches [28.9%]), confirmed or suspected drugs and alcohol (102 searches [22.8%]), tobacco products (54 searches [12.1%]), and pipes and smoking materials (53 searches [11.9%]). Patients who had 1 or more search requests during a substance-related hospital encounter had a statistically significant odds ratio (OR) of having a PDD compared with patients who did not undergo searches (OR, 2.99; 95% CI, 2.32-3.86).

**Conclusions and Relevance:**

In this cohort study of patients with substance-related hospital encounters, most searches did not result in the confiscation of illicit substances, and searches were associated with PDDs. There is an overall need for clear, patient-centered hospital search protocols that support both staff and patient well-being.

## Key Points

**Question:**

How many hospital security searches occur among patients with substance-related encounters and what are search outcomes?

**Findings:**

In this cohort study of 576 hospital room search requests, 79% occurred during substance-related encounters, 44% resulted in nothing confiscated, 29% resulted in confiscation of syringes and unspecified paraphernalia, and 23% in confiscation of illicit drugs or alcohol. Searches were associated with increased odds of patient-directed discharges.

**Meaning:**

These findings suggest that most hospital security searches occurred among patients with substance-related encounters, were more likely to result in confiscating nothing than in confiscating illicit substances, and were associated with patient-directed discharges.

## Teaser

This cohort study evaluates the clinical context and outcomes of room searches among patients with substance-related hospital encounters.

## Introduction

In 2023, hospitals across the US reported that approximately 1 in 5 emergency department (ED) visits and 1 in 4 hospitalizations were associated with drug use.^1^ These ED and hospital encounters offer opportunities to engage patients in substance use disorder (SUD) treatment and harm reduction services.^2,3^ For example, initiating medications for opioid use disorder (MOUD) during hospital encounters is associated with increased addiction treatment postdischarge and decreased readmissions.^4,5^ However, patients with SUDs often face challenges during health care encounters, including stigma and undertreated pain and withdrawal. Limited training along with misconceptions about and negative attitudes toward people with SUDs are prevalent among health care workers and contribute to reluctance to initiate MOUD and adequate pain regimens.^6,7,8,9^ Patients may also be reluctant to disclose substance use due to fear of discrimination and internalized stigma.^10,11^ Consequently, approximately 40% or more of patients with SUDs report continued substance use in hospital settings to manage pain, withdrawal, and/or other negative feelings.^12,13,14,15^

While few hospital systems have guidelines for responding to in-hospital substance use,^16^ one common practice involves security personnel searching patients’ rooms and belongings for illicit substances.^17,18^ Searches are intended to protect patients from overdose and other complications, while also protecting staff (eg, from needle stick injuries).^19,20^ However, qualitative findings in patients with SUDs suggest that searches may contribute to shame and decisions to leave the hospital before completing medical treatments.^12,21,22^ These patient-directed discharges (PDDs) are increasingly common among patients with opioid use disorder (OUD)^23^ and are associated with adverse outcomes, including readmissions and mortality.^24,25,26^

Overall, little is known about hospital searches or their consequences for clinical care. Although qualitative data have identified room searches as a factor in some PDDs, there has yet to be a quantitative description of this association. Our objective was to conduct a descriptive analysis of room searches among patients with substance-related hospital encounters, assessing the clinical context and outcomes after searches, including items confiscated and PDDs.

## Methods

### Study Design

We performed a retrospective cohort study of patients with substance-related hospital encounters at an academic health center using hospital security incident reports and electronic health record (EHR) data. The University of Pennsylvania institutional review board approved our study and did not require informed consent, as data were deidentified, and we followed Strengthening the Reporting of Observational Studies in Epidemiology (STROBE) reporting guidelines.^27^

### Study Setting

The study took place at an urban, academic hospital in Philadelphia, Pennsylvania, with approximately 1000 beds. During the study period, the hospital had published protocols for OUD management, including MOUD initiation and treatment of pain and withdrawal using short-acting opioids. There was also consultative support for patients with SUDs via psychiatry and a specialty pharmacist. While there was no formal policy on managing in-hospital substance use, patients were not permitted to use tobacco products or outside medications, or to possess alcohol, illicit drugs, or drug paraphernalia in the hospital. Per hospital policy, room searches required “good reason,” including “immediate danger, personal safety, exhibiting abnormal or disruptive behavior, medical reasons, obvious impairment or intoxication, [and/or] unlawful acts.” Searches also required patient consent unless staff determined there was an immediate safety concern. Following searches, security disposed of suspected illicit substances and paraphernalia, generally without law enforcement involvement. In addition, clinical staff could add behavioral flags to patients’ EHRs documenting behavior deemed dangerous or disruptive, viewable by anyone looking at the patient’s EHR.

### Data Sources and Population

We obtained EHR data via the Epic Clarity database from July 1, 2021, to July 1, 2023, supplemented by medical record review. We included encounters for patients 18 years and older with a substance-related ED, observation unit, or inpatient hospital admission. We defined substance-related hospital encounters based on inclusion of *International Statistical Classification of Diseases and Related Health Problems, Tenth Revision (ICD-10)* codes consistent with SUDs,^28^ an ED chief concern consistent with drug overdose or withdrawal, and/or a positive verbal screening for active opioid use at ED triage.^29^

To identify visits involving room searches, we used data from an electronic reporting system (Omnigo 2022; Omnigo Software) used by hospital security officers to record staff search requests. Search request reports include request date, whether the search occurred, patient identifiers, professional role of the requesting staff member, and a search description, including location, items confiscated, and, when relevant, PDD occurrence during the security interaction. Some patients had multiple search requests during the same hospitalization, and we counted each search attempt as an independent event in search-level analyses. We then matched the patient identifiers from security reports with EHR data to determine which substance-related hospital encounters involved search requests.

### Measures

We examined outcomes at both the encounter level and search level. Our primary outcomes at the encounter level were search requests and encounter PDDs. Outcomes at the search level included items confiscated and PDDs at the time of searches, which we defined as PDDs documented in security reports. Secondary search outcomes included PDDs later in the hospitalization, naloxone administration for suspected in-hospital drug use later in the hospitalization after searches, and addition of behavioral flags in the EHR documenting searches. We determined that naloxone administration was due to suspected in-hospital drug use if a clinician’s EHR note explicitly stated that use of outside substances most likely prompted the need for naloxone.

Other variables of interest included patient substance use history, complications, and in-hospital SUD treatment. We labeled encounters as involving opioid use if they included any opioid-related criteria (*ICD-10* code consistent with opioid use^30^ and/or positive verbal screening^31^) and the rest as other substance use only. We identified SUD complications, including injection-related infections (*ICD-10* codes for bacteremia, endocarditis, or osteomyelitis^23^) and wounds (indicated by wound care consult placement). We also abstracted receipt of MOUD and/or short-acting opioids before searches among patients with documented opioid use. Other variables included the clinical services on which search requests occurred; the professional role of staff members requesting searches; and the presence of a behavioral flag before searches occurred. Finally, included patient characteristics were age, gender, insurance status, and race and ethnicity, which are recorded in the EHR based on self-report. Race was assessed in this study because prior research^32,33^ has identified racial disparities in other hospital security requests.

### Statistical Analysis

We used descriptive statistics to characterize patient and encounter-level characteristics for substance-related hospital encounters and the clinical context and outcomes of room searches. To compare the distribution of categorical variables across patients and encounters with and without search requests, we performed χ^2^ tests. We also performed Poisson regression to estimate the incidence of a search request and logistic regression to estimate the association between room searches and PDDs, with both models adjusting for patient and encounter covariates. Our logistic regression included search request occurrence as a binary independent variable, and we conducted a sensitivity analysis using the number of search requests as an ordinal variable. Results with a *P* value of less than .05 were defined as statistically significant, and we conducted all analyses in Stata version 18.0 (StataCorp).

## Results

**Figure 1.**
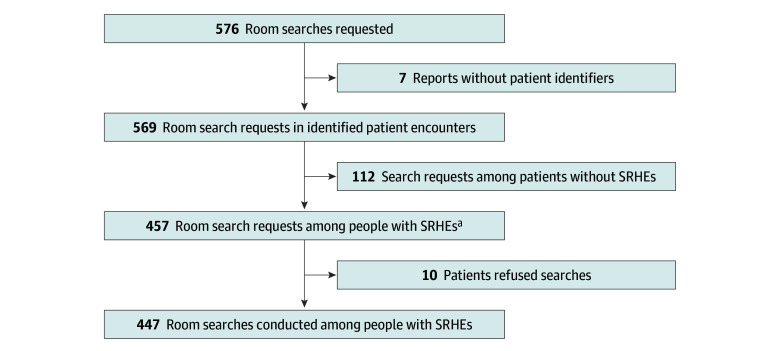
Flow Diagram of Room Search Cohorts ^a^A total of 457 room search requests occurred during 369 unique substance-related hospital encounters (SRHEs) for 283 unique patients.

Between July 2021 and July 2023, there were 13 827 substance-related hospital encounters involving 6985 unique patients (median [IQR] age, 47 [34-58] years; 3863 [55.3%] male; 3688 [52.7%] Black; 302 [4.3%] Hispanic; 2597 [37.2%] White). Over the study period, there were 576 room search requests, among which 457 (79.3%) were requests for searches among patients with substance-related hospital encounters. Ten patients refused searches, so 447 room searches occurred during substance-related hospital encounters (Figure 1). Eight of 10 patients who refused searches had PDDs at the time of search attempts. Some patients had multiple search requests over 1 or more encounters. There were 369 unique substance-related hospital encounters and 283 unique patients with 1 or more search requests.

Compared with patients with substance-related hospital encounters without search requests, a significantly higher proportion of patients with search requests identified as White (180 patients [63.6%] vs 2417 patients [36.1%]), had Medicaid insurance (264 patients [71.5%] vs 7291 patients [54.2%]), and had documented opioid use (246 patients [66.7%] vs 2854 patients [21.2%]) (Table 1). Searches were also more common in those with medical or surgical admissions, injection-related infections, wound care consults, and patients who received MOUDs, short-acting opioids, and in-hospital naloxone. A Poisson regression model adjusting for patient and encounter covariates showed similar results (eTable 1 in Supplement 1).

**Table 1.** Patient- and Encounter-Level Characteristics Comparing All Patients With Substance-Related Hospital Encounters With and Without Room Search Requests

Characteristic	Patients, No. (%) (N = 6985)		*P* value
	With search (n = 283)	Without search (n = 6702)	
Total encounters, No.	369	13 458	NA
Age, y			
18-24	4 (1.4)	746 (11.1)	<.001
25-34	69 (24.4)	1342 (20.0)	
35-44	115 (40.6)	1245 (18.6)	
45-54	50 (17.7)	1084 (16.2)	
55-64	35 (12.4)	1373 (20.5)	
≥65	10 (3.5)	912 (13.6)	
Gender			
Female	135 (47.7)	2986 (44.6)	.57
Male	148 (52.3)	3715 (55.4)	
Other	0	1 (0.01)	
Race			
Black	80 (28.3)	3608 (53.8)	<.001
Unknown or another race^a^	23 (8.1)	677 (10.1)	
White	180 (63.6)	2417 (36.1)	
Ethnicity			
Hispanic or Latine	15 (5.3)	287 (4.3)	.23
Not Hispanic or Latine	267 (94.4)	6319 (94.3)	
Not reported	1 (0.4)	96 (1.4)	
Insurance status			
Commercial	57 (15.5)	2853 (21.2)	<.001
Medicaid	264 (71.5)	7291 (54.2)	
Medicare	44 (11.9)	3044 (22.6)	
Self-pay	4 (1.1)	270 (2.0)	
Service type			
Emergency	77 (20.9)	7250 (53.9)	<.001
Medical	235 (63.7)	4247 (31.6)	
Surgical	46 (12.5)	1288 (9.6)	
Other (observation unit, OB/GYN, ICU, Neurology)	11 (3.0)	673 (5.0)	
Substance use type			
Opioid use	246 (66.7)	2854 (21.2)	<.001
Other substance use only	123 (33.3)	10 604 (78.8)	
MOUD receipt	180 (48.8)	1226 (9.1)	<.001
Short-acting opioid receipt	231 (62.6)	3944 (29.3)	<.001
Naloxone receipt	49 (13.3)	277 (2.1)	<.001
Injection-related infections	92 (24.9)	652 (4.8)	<.001
Wound care consult	54 (14.6)	324 (2.4)	<.001

Abbreviations: ICU, intensive care units; MOUD, medications for opioid use disorder, including buprenorphine and methadone; NA, not applicable; OB/GYN, obstetrics and gynecology.

^a^ Other patient-reported races included American Indian or Alaskan Native, Asian, East Indian, and Native Hawaiian or Other Pacific Islander.

Table 2 describes the clinical context and outcomes of the 457 room search requests. The most common clinical services for search requests were emergency medicine and medical services, and 376 searches (82.3%) were requested by nurses. A total of 320 searches (70.0%) occurred in patients with documented opioid use, and among those with opioid use, 84 patients (26.3%) had received neither MOUD nor short-acting opioids before searches.

**Table 2.** Clinical Context of Room Search Requests

Context characteristic	Search requests, No. (%)
Service type (n = 457)	
Emergency	187 (40.9)
Medical	184 (40.3)
Surgical specialties	53 (11.6)
Other (observation unit, OB/GYN, ICU, neurology)	33 (7.2)
Role of staff members requesting searches (n = 457)	
Nurse	376 (82.3)
Attending physician	26 (5.7)
Resident physician	8 (1.8)
Other clinical staff	6 (1.3)
Security officer	2 (0.4)
Unknown	39 (8.5)
Medications received by time of search request among patients with documented opioid use (n = 320)	
Methadone or buprenorphine plus short-acting opioids	126 (39.4)
Methadone or buprenorphine (without short-acting opioids)	50 (15.6)
Short-acting opioids (without methadone or buprenorphine)	60 (18.8)
Neither MOUD nor short-acting opioids	84 (26.3)
Patient-directed discharges (n = 457)	
Completed at time of search attempt	20 (4.4)
Completed later in hospitalization	159 (34.8)
Naloxone administration due to suspected illicit opioid overdose (n = 457)	
Before search request	21 (4.7)
After search, later in hospitalization	7 (1.6)
Behavioral flag presence in electronic medical record (n = 457)	
Before search request	122 (26.7)
Describing search	46 (10.1)

Abbreviations: ICU, intensive care units; MOUD, medications for opioid use disorder, including buprenorphine and methadone; OB/GYN, obstetrics and gynecology.

Twenty-one searches (4.7%) were requested after patients required naloxone for suspected in-hospital opioid overdose, and 7 patients (1.6%) subsequently required naloxone for suspected in-hospital drug use later in the hospital encounter (Table 2). Among patients with substance-related encounters and search requests, there were 122 behavioral flags present in patient medical records before search requests (122 flags [26.7%]), and 46 behavioral flags added to medical records documenting the search attempt (following 10.1% of requests). There were also 20 PDDs (4.4%) at the time of completed or attempted searches, and 159 patients (34.8%) had PDDs later in the hospitalization.

**Figure 2.**
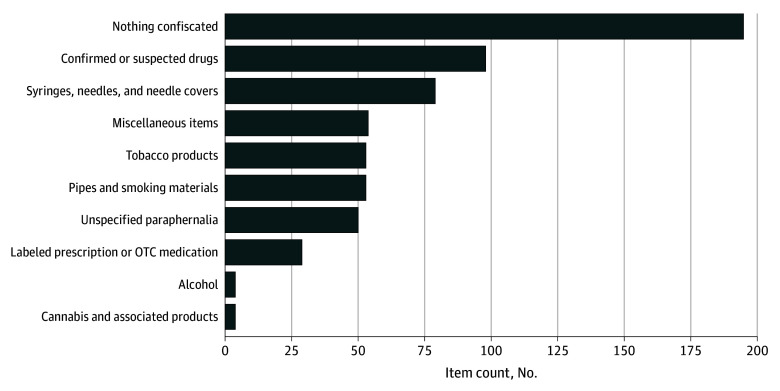
Items Confiscated During Completed Searches There were 447 total completed searches. The sum of items found is greater than 447, as multiple items were found in some searches. OTC indicates over the counter.

In 195 completed searches (43.6%), nothing was confiscated (Figure 2). There was otherwise confiscation of syringes, needles, needle covers, and unspecified paraphernalia in 129 searches (28.9%), confirmed or suspected drugs in 98 (21.9%), miscellaneous items that were not identified as substance-related in 54 (12.1%), and additional items, as specified in Figure 2 (see eTable 3 in Supplement 1 for miscellaneous item list). Of the 21 searches requested following suspected in-hospital overdose, 9 resulted in nothing found, 6 in confiscation of suspected illicit drugs or alcohol, 3 in confiscation of tobacco products, and 2 in confiscation of syringes or unspecified paraphernalia. Among the 7 patients requiring naloxone for suspected in-hospital overdose after searches, there was confiscation of suspected illicit drugs in 3 searches, syringes in 3, and a razor in 1.

Finally, we examined the association between room searches and PDDs using multivariable logistic regression (Table 3). After adjustment for patient and clinical characteristics, patients with 1 or more search requests during a given encounter were significantly more likely to have a PDD compared with patients who did not undergo searches (adjusted odds ratio, 2.99; 95% CI, 2.32-3.86). Results were similar in a sensitivity analysis using the number of search requests as an ordinal variable (eTable 2 in Supplement 1).

**Table 3.** Adjusted Model for Association Between Room Searches and Patient-Directed Discharges During Substance-Related Hospital Encounters

Characteristic	Adjusted odds ratio (95% CI)	*P* value
≥1 Searches	2.99 (2.32-3.86)	<.001
Age		
18-24	1 [Reference]	NA
25-34	2.14 (1.54-2.98)	<.001
35-44	2.20 (1.58-3.06)	<.001
45-54	1.62 (1.15-2.26)	.01
55-64	1.50 (1.07-2.11)	.02
≥65	1.05 (0.69-1.61)	.80
Gender		
Female	1 [Reference]	NA
Male	1.52 (1.34-1.74)	<.001
Race		
Black	1 [Reference]	NA
Unknown or another race^a^	0.69 (0.51-0.91)	.01
White	1.39 (1.21-1.60)	<.001
Ethnicity		
Hispanic or Latine	1 [Reference]	NA
Not Hispanic or Latine	0.92 (0.66-1.28)	.63
Not reported	1.78 (0.88-3.59)	.11
Insurance status		
Commercial	1 [Reference]	NA
Medicaid	1.49 (1.25-1.77)	<.001
Medicare	0.84 (0.66-1.06)	.14
Self-pay	1.75 (1.15-2.67)	.009
Service type		
Emergency	1 [Reference]	NA
Medical	1.36 (1.17-1.58)	<.001
Other (observation unit, OB/GYN, ICU, Neurology)	0.73 (0.51-1.03)	.07
Surgical	0.36 (0.25-0.52)	<.001
Substance use type		
Opioid use	1 [Reference]	NA
Other substance use only	0.59 (0.51-0.68)	<.001
MOUD receipt	1.10 (0.91-1.34)	.32
Short-acting opioid receipt	0.98 (0.84-1.15)	.81
Naloxone receipt	0.82 (0.55-1.21)	.31
Wound care consult	0.81 (0.57-1.14)	.23
Behavioral flag	3.54 (2.66-4.69)	<.001
Injection-related infections	1.53 (1.22-1.92)	<.001

Abbreviations: ICU, intensive care units; MOUD, medications for opioid use disorder, including buprenorphine and methadone; NA, not applicable; OB/GYN, obstetrics and gynecology.

^a^ Other patient-reported races included American Indian or Alaskan Native, Asian, East Indian, and Native Hawaiian or Other Pacific Islander.

## Discussion

This study is among the first to examine hospital security searches, offering insights into the application and outcomes of a common hospital policy. Over 2 years at an academic hospital, most searches occurred among patients with substance-related encounters. Confiscation of illicit drugs and alcohol happened in a minority of searches, and more frequently, security confiscated nothing. In addition, searches did not prevent all suspected in-hospital opioid overdoses, and there was a significant association between search attempts and PDDs. Overall, our findings highlight important considerations for room searches among patients with substance-related hospital encounters, including implications for hospital policies aiming to balance patient and clinician safety.

First, our study sheds light on potential risks and benefits of room searches during substance-related hospitalizations. Prior work has shown that without clear, evidence-based guidelines, responses to suspected in-hospital substance use, including room searches, may vary based on clinicians’ beliefs about drug use.^14^ In our study, about 1 in 5 searches resulted in confiscation of suspected drugs, and there were few naloxone administrations following room searches (1.6%). However, almost half of searches resulted in nothing found, and suspected in-hospital overdoses still occurred after searches. Previous research suggests that increased monitoring of patients with SUDs does not always prevent in-hospital substance use and may lead to riskier behaviors, such as use in locked bathrooms.^34^ In nonhospital settings, limiting access to syringes and other drug-related equipment can also increase injection-related harms, including infectious disease transmission.^35,36^ Importantly, this study does not evaluate possible benefits of searches for staff, such as decreasing needle stick injuries or injuries from miscellaneous items, nor does it evaluate the impact of confiscating supplies on drug use practices after hospitalization. Nevertheless, our findings present early evidence to inform discussions weighing the complex risks and benefits of room search practices.

Our findings also add to previous qualitative studies highlighting the risk of PDDs following security searches.^13,21^ Patients who underwent search attempts in our study were approximately 3 times as likely to have PDDs compared with those who did not undergo searches. While we cannot verify that searches caused PDDs, nor eliminate all confounding factors, searches may have contributed to experiences of stigma and bias for patients, impacting decisions to leave. Prior research has also demonstrated an association between PDDs and undertreatment of pain and withdrawal during opioid-related hospitalizations.^37^ In our study, over 1 in 4 patients with documented opioid use had not received MOUDs or other opioid agonists before searches. While this may have been appropriate in some cases, it is also possible that inadequate pain and withdrawal management contributed to in-hospital substance use, triggering a search. Future work should focus on best practices to address unmanaged pain and withdrawal to potentially prevent and respond to in-hospital substance use.

Our results also have important implications for room search policies and for strategies to mitigate in-hospital substance use. Implementing a structured assessment, such as the Hospital Misuse Checklist,^38^ to determine the likelihood of in-hospital substance use could reduce the number of unnecessary searches. Another hospital has implemented protocols to reduce stigma and searches for patients with SUDs by explaining substance use policies to all patients, allowing patients to safely discard or store prohibited items until discharge, and assessing pain and withdrawal needs proactively.^16^ Our findings also demonstrate the importance of involving nurses in development of policies around searches and in-hospital substance use. In this study, nurses requested most searches. As the health care professionals who spend the most time with patients,^39^ nurses may be the first to notice in-hospital substance use and be at highest risk for negative experiences when patients use drugs.^20,40^ Our data suggest the need to incorporate nursing perspectives into pathways for responding to suspected substance use in hospital settings. Moreover, training in addiction care across health professions can increase staff preparedness to care for patients with SUDs.^41,42^ Recently, efforts to expand SUD training for nurses have included incorporating addiction care into nursing school curricula and establishing addiction nursing fellowships and certificate programs.^43,44^ Future research could evaluate whether these efforts decrease in-hospital substance use and security searches.

When searches do occur, additional measures should aim to minimize impact on subsequent patient care, including standardizing search documentation. In our study, over one quarter of patients had behavioral flags in their medical records before searches and others had behavioral flags placed in their medical records documenting searches. Although intended to prompt safety precautions for staff, behavioral flags have previously been associated with disparities in care.^45^ Therefore, implementing defined, narrow criteria for behavioral flags or other potentially stigmatizing medical record documentation might help decrease future bias in care.

More broadly, hospitals should explore ways to reduce bias and stigma for patients who undergo searches, including incorporating patient perspectives. Hospital policies affecting patients with SUDs have often not involved patient input or evidence-based practices.^46^ Despite the broad criteria allowing for security searches at the hospital in this study, searches primarily impacted people with documented substance use. Future work should include patient perspectives to develop best search practices that minimize trauma for patients and promote safety for patients and staff.

Finally, in contrast to prior research, White patients with substance-related hospital encounters were more likely to undergo searches compared with Black patients in our study. In previous studies looking at a general hospital population, hospital staff called security more frequently for Black patients and their visitors than for patients of other races.^47,48^ Our differing results may relate to demographic differences in drug use behaviors. Room searches were more common among patients with complications of injection drug use, and recent data suggest that White patients may be more likely to inject opioids than are Black patients.^49^ MOUD receipt was also associated with search requests, and Black patients have been shown to be less likely to receive MOUD compared with White patients.^50^ In previous studies, Black patients have described that experiences of stigma and racism have led to hesitation in disclosing substance use during health care encounters.^51,52,53^ Consequently, underrecognition of SUDs in Black patients may also contribute to our findings about search requests. As health care systems work to reduce inequities in SUD care, we need to consider how responses to in-hospital substance use intersect with experiences of stigma and discrimination that patients with SUDs from minoritized groups may face.

### Limitations

This study had limitations. We described outcomes from a single hospital in a city with a high SUD prevalence,^54^ so findings may not generalize to other settings. In addition, security incident reports’ identification of drugs and paraphernalia may not be accurate, as we do not know patients’ intended use of items or the relevance of miscellaneous items found. We also had incomplete documentation about the stated reasons for searches, including specific concerns prompting searches, and some search reports had missing data. In addition, we used EHR data to identify our sample of people who use drugs, which may not fully capture all people who use substances, especially patients from marginalized groups.^55^ While the study hospital also had an ED triage screening for patient-reported opioid use, there was not a similar screening for other illicit substance use, so we may have missed potential patients. Additionally, our measure of naloxone administration for suspected in-hospital drug use relied on documented clinician judgment, which is subject to bias and may not reflect true incidence of in-hospital overdose.

## Conclusions

In this cohort study of patients with substance-related hospital encounters, most security searches of patients did not result in the confiscation of drugs or alcohol, and searches were associated with PDDs. Hospital systems may support positive outcomes for both patients and staff by developing clear policies around in-hospital substance use and searches that incorporate continued evaluation of search risks and benefits, evidence-based prevention of in-hospital substance use, and perspectives from patients, nurses, and experts in addiction care.

## Notes

Supplement 1. **eTable 1. **Association Between Encounter-Level Characteristics and Presence of a Search Request During Substance-Related Hospital Encounters **eTable 2. **Adjusted Model for Association Between Room Search Number and Patient-Directed Discharges During Substance-Related Hospital Encounters **eTable 3. **Miscellaneous Items Found During Completed Searches

Supplement 2.
Data Sharing Statement

## References

[1] U.S. Centers for Disease Control and Prevention, National Center for Health Statistics. Hospital encounters involving drug use by month from selected hospitals. September 3, 2024. Accessed September 10, 2024. https://www.cdc.gov/nchs/dhcs/drug-use/drug-use.htm

[2] Weinstein ZM, Englander H. Reachable Moment: Hospital-Based Interventions. In: Wakeman SE, Rich JD, eds. Treating Opioid Use Disorder in General Medical Settings. Springer International Publishing; 2021:43-56. doi:10.1007/978-3-030-80818-1_4

[3] Nordeck CD, Welsh C, Schwartz RP, . Rehospitalization and substance use disorder (SUD) treatment entry among patients seen by a hospital SUD consultation-liaison service. Drug Alcohol Depend. 2018;186:23-28. doi:10.1016/j.drugalcdep.2017.12.043 29529456 PMC5922267

[4] Mancher M, Leshner AI, eds. National Academies of Sciences, Engineering, and Medicine; Health and Medicine Division; Board on Health Sciences Policy; Committee on Medication-Assisted Treatment for Opioid Use Disorder. Medications for Opioid Use Disorder Save Lives. National Academies Press; 2019. Accessed March 19, 2024. https://www.ncbi.nlm.nih.gov/books/NBK538936/30896911

[5] O’Rourke BP, Hogan TH, Teater J, . Initiation of medication for opioid use disorder across a health system: a retrospective analysis of patient characteristics and inpatient outcomes. Drug Alcohol Depend Rep. 2022;5:100114. doi:10.1016/j.dadr.2022.100114 36844164 PMC9948916

[6] van Boekel LC, Brouwers EPM, van Weeghel J, Garretsen HFL. Stigma among health professionals towards patients with substance use disorders and its consequences for healthcare delivery: systematic review. Drug Alcohol Depend. 2013;131(1-2):23-35. doi:10.1016/j.drugalcdep.2013.02.018 23490450

[7] Dickson-Gomez J, Spector A, Weeks M, Galletly C, McDonald M, Green Montaque HD. “You’re not supposed to be on it forever”: medications to treat opioid use disorder (MOUD) related stigma among drug treatment providers and people who use opioids. Subst Abuse. Published online June 27, 2022. doi:10.1177/11782218221103859 35783464 PMC9243471

[8] Becker TD, Eschliman EL, Thakrar AP, Yang LH. A conceptual framework for how structural changes in emerging acute substance use service models can reduce stigma of medications for opioid use disorder. Front Psychiatry. 2023;14:1184951. doi:10.3389/fpsyt.2023.1184951 37829763 PMC10565357

[9] Brezing C, Marcovitz D. Stigma and Persons with Substance Use Disorders. In: Parekh R, Childs EW, eds. Stigma and Prejudice: Touchstones in Understanding Diversity in Healthcare. Springer International Publishing; 2016:113-132. doi:10.1007/978-3-319-27580-2_7.

[10] Paris R, Herriott AL, Maru M, Hacking SE, Sommer AR. Secrecy versus disclosure: women with substance use disorders share experiences in help seeking during pregnancy. Matern Child Health J. 2020;24(11):1396-1403. doi:10.1007/s10995-020-03006-1 33025236

[11] Earnshaw VA, Bogart LM, Menino D, . Disclosure, stigma, and social support among young people receiving treatment for substance use disorders and their caregivers: a qualitative analysis. Int J Ment Health Addict. 2019;17(6):1535-1549. doi:10.1007/s11469-018-9930-8 33312084 PMC7731618

[12] McNeil R, Small W, Wood E, Kerr T. Hospitals as a ‘risk environment’: an ethno-epidemiological study of voluntary and involuntary discharge from hospital against medical advice among people who inject drugs. Soc Sci Med. 2014;105:59-66. doi:10.1016/j.socscimed.2014.01.010 24508718 PMC3951660

[13] Ti L, Voon P, Dobrer S, Montaner J, Wood E, Kerr T. Denial of pain medication by health care providers predicts in-hospital illicit drug use among individuals who use illicit drugs. Pain Res Manag. 2015;20(2):84-88. doi:10.1155/2015/868746 25562839 PMC4391443

[14] Strike C, Robinson S, Guta A, . Illicit drug use while admitted to hospital: patient and health care provider perspectives. PLoS One. 2020;15(3):e0229713. doi:10.1371/journal.pone.0229713 32134973 PMC7058273

[15] Parmar GS, Hayashi K, Nolan S, . Non-medical prescription opioid use and in-hospital illicit drug use among people who use drugs. Drug Alcohol Rev. 2021;40(6):959-963. doi:10.1111/dar.13246 33543535 PMC8333188

[16] Martin M, Snyder HR, Otway G, Holpit L, Day LW, Seidman D. In-hospital substance use policies: an opportunity to advance equity, reduce stigma, and offer evidence-based addiction care. J Addict Med. 2023;17(1):10-12. doi:10.1097/ADM.0000000000001046 35914181 PMC9897266

[17] Burnette AT. Searches of hospital patients, their rooms and belongings. Health Care Law Monthly. October 2012. Accessed August 16, 2023. https://www.alston.com/-/media/files/insights/publications/2012/10/searches-of-hospital-patients-their-rooms-and--bel/files/hcmonthlyoct2012burnette/fileattachment/hcmonthlyoct2012burnette.pdf 23134041

[18] Lennox R, Martin L, Brimner C, O’Shea T. Hospital policy as a harm reduction intervention for people who use drugs. Int J Drug Policy. 2021;97:103324. doi:10.1016/j.drugpo.2021.103324 34153628

[19] Huxley-Reicher Z, Puglisi LB, Tetrault JM, . Response to substance use during hospitalization: a survey study of current and ideal policies and practices. J Hosp Med. 18(9):829-834. doi:10.1002/jhm.13162 37475186

[20] Horner G, Daddona J, Burke DJ, Cullinane J, Skeer M, Wurcel AG. “You’re kind of at war with yourself as a nurse”: perspectives of inpatient nurses on treating people who present with a comorbid opioid use disorder. PLoS One. 2019;14(10):e0224335. doi:10.1371/journal.pone.0224335 31648259 PMC6812769

[21] Pollini RA, Paquette CE, Drvar T, . A qualitative assessment of discharge against medical advice among patients hospitalized for injection-related bacterial infections in West Virginia. Int J Drug Policy. 2021;94:103206. doi:10.1016/j.drugpo.2021.103206 33765516 PMC8373672

[22] Simon R, Snow R, Wakeman S. Understanding why patients with substance use disorders leave the hospital against medical advice: a qualitative study. Subst Abus. 2020;41(4):519-525. doi:10.1080/08897077.2019.1671942 31638862

[23] Thakrar AP, Lowenstein M, Greysen SR, Delgado MK. Trends in before medically advised discharges for patients with opioid use disorder, 2016-2020. JAMA. 2023;330(23):2302-2304. doi:10.1001/jama.2023.21288 38048121 PMC10696509

[24] Appa A, Adamo M, Le S, . Patient-directed discharges among persons who use drugs hospitalized with invasive staphylococcus aureus infections: opportunities for improvement. Am J Med. 2022;135(1):91-96. doi:10.1016/j.amjmed.2021.08.007 34508704 PMC11552656

[25] Glasgow JM, Vaughn-Sarrazin M, Kaboli PJ. Leaving against medical advice (AMA): risk of 30-day mortality and hospital readmission. J Gen Intern Med. 2010;25(9):926-929. doi:10.1007/s11606-010-1371-4 20425146 PMC2917668

[26] Southern WN, Nahvi S, Arnsten JH. Increased risk of mortality and readmission among patients discharged against medical advice. Am J Med. 2012;125(6):594-602. doi:10.1016/j.amjmed.2011.12.017 22513194 PMC3372411

[27] von Elm E, Altman DG, Egger M, Pocock SJ, Gøtzsche PC, Vandenbroucke JP; STROBE Initiative. The Strengthening the Reporting of Observational Studies in Epidemiology (STROBE) statement: guidelines for reporting observational studies. Lancet. 2007;370(9596):1453-1457. doi:10.1016/S0140-6736(07)61602-X 18064739

[28] Rowe CL, Santos GM, Kornbluh W, Bhardwaj S, Faul M, Coffin PO. Using ICD-10-CM codes to detect illicit substance use: a comparison with retrospective self-report. Drug Alcohol Depend. 2021;221:108537. doi:10.1016/j.drugalcdep.2021.108537 33621806 PMC11008535

[29] Lowenstein M, McFadden R, Abdel-Rahman D, . Redesign of opioid use disorder screening and treatment in the ED. NEJM Catal Innov Care Deliv. 2022;3(1). doi:10.1056/CAT.21.0297 37961066 PMC10641724

[30] Owens PL, Weiss AJ, Barrett ML. Table 2, ICD-10-CM diagnosis codes defining opioid-related inpatient stays. May 26, 2020. Accessed June 17, 2024. https://www.ncbi.nlm.nih.gov/books/NBK559382/table/sb258.tab4/

[31] Lowenstein M, Perrone J, McFadden R, . Impact of universal screening and automated clinical decision support for the treatment of opioid use disorder in emergency departments: a difference-in-differences analysis. Ann Emerg Med. 2023;82(2):131-144. doi:10.1016/j.annemergmed.2023.03.033 37318434 PMC11019868

[32] Green CR, McCullough WR, Hawley JD. Visiting Black patients: racial disparities in security standby requests. J Natl Med Assoc. 2018;110(1):37-43. doi:10.1016/j.jnma.2017.10.009 29510841

[33] Valtis YK, Stevenson KE, Murphy EM. Race and ethnicity and the utilization of security responses in a hospital setting. J Gen Intern Med. 2023;38(1):30-35. doi:10.1007/s11606-022-07525-1 35556213 PMC9849525

[34] Biancarelli DL, Biello KB, Childs E, . Strategies used by people who inject drugs to avoid stigma in healthcare settings. Drug Alcohol Depend. 2019;198:80-86. doi:10.1016/j.drugalcdep.2019.01.037 30884432 PMC6521691

[35] Walker S, Seear K, Higgs P, Stoové M, Wilson M. “A spray bottle and a lollipop stick”: an examination of policy prohibiting sterile injecting equipment in prison and effects on young men with injecting drug use histories. Int J Drug Policy. 2020;80:102532. doi:10.1016/j.drugpo.2019.07.027 31427211

[36] Werb D, Wood E, Small W, . Effects of police confiscation of illicit drugs and syringes among injection drug users in Vancouver. Int J Drug Policy. 2008;19(4):332-338. doi:10.1016/j.drugpo.2007.08.004 17900888 PMC2529170

[37] Santos CJ, Shofer FS, Lowenstein M, Perrone J. Discharges “against medical advice” in patients with opioid-related hospitalizations. J Addict Med. 2021;15(1):49-54. doi:10.1097/ADM.0000000000000688 32541363

[38] Mitchell LM, Milliken A, Montgomery MW, Singh SK, Suzuki J. The opioid crisis and the inpatient floor: considering injection drug use in the management of infective endocarditis and acute pain. Harv Rev Psychiatry. 2020;28(5):334-340. doi:10.1097/HRP.0000000000000259 32453063 PMC7492483

[39] Smiley RA, Allgeyer RL, Shobo Y, . The 2022 national nursing workforce survey. J Nurs Regul. 2023;14(1):S1-S90. doi:10.1016/S2155-8256(23)00047-937012978

[40] Ford R. Interpersonal challenges as a constraint on care: the experience of nurses’ care of patients who use illicit drugs. Contemp Nurse. 2011;37(2):241-252. doi:10.5172/conu.2011.37.2.241 21692595

[41] Ayu AP, van der Ven M, Suryani E, . Improving medical students’ attitude toward patients with substance use problems through addiction medicine education. Subst Abus. 2022;43(1):47-55. doi:10.1080/08897077.2020.1732512 32105582

[42] Wakeman SE, Kanter GP, Donelan K. Institutional substance use disorder intervention improves general internist preparedness, attitudes, and clinical practice. J Addict Med. 2017;11(4):308-314. doi:10.1097/ADM.0000000000000314 28375881

[43] Fox JM, Wason K, Beers D, . The creation of an addiction nursing fellowship program for registered nurses: a unique approach to enhancing the addiction-treatment workforce. Subst Abus. 2023;44(1):24-31. doi:10.1177/08897077231169566 37226903

[44] DeBrew J. “Looks can be deceiving”: an innovative way to teach nursing students about substance use disorder. Creat Nurs. 2023;29(1):141-156. doi:10.1177/107845352202900102 37550995

[45] Agarwal AK, Seeburger E, O’Neill G, . Prevalence of behavioral flags in the electronic health record among Black and White patients visiting the emergency department. JAMA Netw Open. 2023;6(1):e2251734. doi:10.1001/jamanetworkopen.2022.51734 36656576 PMC9857105

[46] Winetsky D, Weinrieb RM, Perrone J. Expanding treatment opportunities for hospitalized patients with opioid use disorders. J Hosp Med. 2018;13(1):62-64. doi:10.12788/jhm.2861 29073311

[47] Green CR, McCullough WR, Hawley JD. Visiting Black patients: racial disparities in security standby requests. J Natl Med Assoc. 2018;110(1):37-43. doi:10.1016/j.jnma.2017.10.009 29510841

[48] Valtis YK, Stevenson KE, Murphy EM, . Race and ethnicity and the utilization of security responses in a hospital setting. J Gen Intern Med. 2023;38(1):30-35. doi:10.1007/s11606-022-07525-1 35556213 PMC9849525

[49] Karandinos G, Unick J, Ondocsin J, . Decrease in injection and rise in smoking and snorting of heroin and synthetic opioids, 2000-2021. Drug Alcohol Depend. 2024;263:111419. doi:10.1016/j.drugalcdep.2024.111419 39216201 PMC11684856

[50] Entress RM. The intersection of race and opioid use disorder treatment: a quantitative analysis. J Subst Abuse Treat. 2021;131:108589. doi:10.1016/j.jsat.2021.108589 34426022

[51] Khatri UG, Nguemeni Tiako MJ, Gebreyesus A, Reid A, Jacoby SF, South EC. “A lack of empathy:” a qualitative study of Black people seeking treatment for opioid use disorder. SSM Qual Res Health. 2023;4:100298. doi:10.1016/j.ssmqr.2023.100298

[52] Mays VM, Jones AL, Delany-Brumsey A, Coles C, Cochran SD. Perceived discrimination in health care and mental health/substance abuse treatment among Blacks, Latinos, and Whites. Med Care. 2017;55(2):173-181. doi:10.1097/MLR.0000000000000638 27753743 PMC5233585

[53] Wood E, Elliott M. Opioid Addiction Stigma: The Intersection of Race, Social Class, and Gender. Subst Use Misuse. 2020;55(5):818-827. doi:10.1080/10826084.2019.1703750 31868067

[54] Philadelphia Department of Public Health. Division of Substance Use Prevention and Harm Reduction 2021 annual report. 2021. Accessed February 5, 2025. https://www.phila.gov/documents/2020-annual-report-of-the-division-of-suphr/

[55] Williams EC, Fletcher OV, Frost MC, Harris AHS, Washington DL, Hoggatt KJ. Comparison of substance use disorder diagnosis rates from electronic health record data with substance use disorder prevalence rates reported in surveys across sociodemographic groups in the Veterans Health Administration. JAMA Netw Open. 2022;5(6):e2219651. doi:10.1001/jamanetworkopen.2022.19651 35771574 PMC9247731

