# Supplementary material for: Hospital Security Searches Among Patients With Substance-Related Encounters
Source: JAMA Netw Open. 2025 Mar 18;8(3):e251068. doi: 10.1001/jamanetworkopen.2025.1068 (PMC11920837; doi:10.1001/jamanetworkopen.2025.1068)
Supplement: Supplement 1. — eTable 1. Association Between Encounter-Level Characteristics and Presence of a Search Request During Substance-Related Hospital Encounters eTable 2. Adjusted Model for Association Between Room Search Number and Patient-Directed Discharges During Substance-Related Hospital Encounters eTable 3. Miscellaneous Items Found During Completed Searches [file jamanetwopen-e251068-s001.pdf]

Supplemental Online Content

Nessen S, Thakrar A, Perrone J, Xu L, McFadden R, Lowenstein M. Hospital security searches among patients with substance-related encounters. *JAMA Netw Open*. 2025;8(3):e251068. doi:10.1001/jamanetworkopen.2025.1068

eTable 1. Association Between Encounter-Level Characteristics and Presence of a Search Request During Substance-Related Hospital Encounters

eTable 2. Adjusted Model for Association Between Room Search Number and Patient-Directed Discharges During Substance-Related Hospital Encounters

eTable 3. Miscellaneous Items Found During Completed Searches

This supplemental material has been provided by the authors to give readers additional information about their work.

© 2025 Nessen S et al. *JAMA Network Open*.

eTable 1. Association Between Encounter-Level Characteristics and Presence of a Search Request During Substance-Related Hospital Encounters

Covariate	IRR (95% CI)	*P* value
**Age**		
18-24	1 [Reference]	
25-34	3.19 (1.39-7.34)	0.006
35-44	3.39 (1.48-7.78)	0.004
45-54	1.96 (0.84-4.60)	0.12
55-64	1.74 (0.73-4.13)	0.21
>=65	0.79 (0.27-2.27)	0.66
**Gender**		
Female	1 [Reference]	
Male	0.83 (0.67-1.03)	0.08
Other	<0.001	0.99
**Race**		
Black	1 [Reference]	
White	1.73 (1.32-2.26)	<0.001
Unknown or another race [a]	1.39 (0.88-2.19)	0.16
**Ethnicity**		
Hispanic or Latine	1 [Reference]	
Not Hispanic or Latine	0.91 (0.57-1.46)	0.70
Not Reported	0.32 (0.04-2.36)	0.26
**Insurance status**		
Commercial	1 [Reference]	
Medicaid	1.47 (1.09-1.98)	0.01
Medicare	0.95 (0.62-1.44)	0.80
Self-pay	1.48 (0.53-4.10)	0.45
**Service type**		
Emergency	1 [Reference]	
Medical	2.43 (1.77-3.33)	<0.001
Other (Observation unit, OB/GYN [b], ICU [c], Neurology)	1.16 (0.60-2.23)	0.66
Surgical	2.11 (1.35-3.29)	0.001
**Substance use type**		
Opioid use	1 [Reference]	
Other	0.36 (0.28-0.47)	<0.001
**MOUD [d] receipt**	1.69 (1.30-2.19)	<0.001
**Short-acting opioid receipt**	1.44 (1.11-1.88)	0.007
**Naloxone receipt**	1.87 (1.35-2.60)	<0.001

© 2025 Nessen S et al. *JAMA Network Open*.

**Serious injection related infections**	1.53 (1.17-1.99)	0.002
**Wound care consult**	1.59 (1.17-2.17)	0.003

[a] Other patient-reported races included American Indian or Alaskan Native, Asian, East Indian, and Native Hawaiian or Other Pacific Islander

[b] OB/GYN = obstetrics and gynecology

[c] ICU = intensive care units

[d] MOUD = medications for opioid use disorder, including buprenorphine and methadone

© 2025 Nessen S et al. *JAMA Network Open*.

eTable 2. Adjusted Model for Association Between Room Search Number and Patient-Directed Discharges During Substance-Related Hospital Encounters

	Adjusted Odds ratio (95% CI)	*P* value
**Search number**	2.18 (1.79-2.65)	<0.001
**Age**		
18-24	1 [Reference]	
25-34	2.17 (1.56- 3.01)	<0.001
35-44	2.23 (1.60-3.10)	<0.001
45-54	1.64 (1.17-2.29)	0.004
55-64	1.52 (1.08-2.14)	0.02
65 and up	1.06 (0.69-1.61)	0.80
**Gender**		
Female	1 [Reference]	
Male	1.52 (1.33-1.74)	<0.001
**Race**		
Black	1 [Reference]	
Unknown or another race [a]	0.68 (0.51-0.91)	0.01
White	1.39 (1.21-1.60)	<0.001
**Ethnicity**		
Hispanic or Latine	1 [Reference]	
Not Hispanic or Latine	0.91 (0.65-1.27)	0.58
Not Reported	1.76 (0.87-3.54)	0.11
**Insurance status**		
Commercial	1 [Reference]	
Medicaid	1.48 (1.25-1.76)	<0.001
Medicare	0.83 (0.65-1.05)	0.12
Self-pay	1.75 (1.15-2.67)	0.009
**Service type**		
Emergency	1 [Reference]	
Medical	1.36 (1.17-1.58)	<0.001
Surgical	0.36 (0.25-0.52)	<0.001
Other (Observation unit, OB/GYN [b], ICU [c], Neurology)	0.73 (0.51-1.04)	0.08
**Substance use type**		
Opioid use	1 [Reference]	
Other substance use only	0.58 (0.50-0.67)	<0.001
**MOUD [d] receipt**	1.09 (0.90-1.32)	0.40
**Short-acting opioid receipt**	0.98 (0.84-1.15)	0.81

© 2025 Nessen S et al. *JAMA Network Open*.

**Naloxone receipt**	0.82 (0.55-1.21)	0.32
**Wound care consult**	0.81 (0.57-1.15)	0.25
**Behavioral flag**	3.56 (2.68-4.72)	<0.001
**Injection-related infections**	1.50 (1.20-1.89)	<0.001

[a] Other patient-reported races included American Indian or Alaskan Native, Asian, East Indian, and Native Hawaiian or Other Pacific Islander

[b] OB/GYN = obstetrics and gynecology

[c] ICU = intensive care units

[d] MOUD = medications for opioid use disorder, including buprenorphine and methadone

© 2025 Nessen S et al. *JAMA Network Open*.

eTable 3. Miscellaneous Items Found During Completed Searches

**Item found**	**Count** N=54 (%)
aluminum foil, paper	2 (3.7)
bullet shell	1 (1.9)
copper wire	1 (1.9)
eating utensils	2 (3.7)
empty containers	9 (16.7)
knife	7 (13.0)
medical supplies	1 (1.9)
small tubes and rods (straws, hollow pens)	5 (9.3)
nail-related items (files, clippers)	6 (11.1)
pepper spray	4 (7.4)
other sharp items (razors, scissors, sowing needles)	9 (16.7)
tools (wrench, screwdriver)	4 (7.4)
small metal cap	1 (1.9)
vials of saline	1 (1.9)
wooden stick	1 (1.9)

© 2025 Nessen S et al. *JAMA Network Open*.
