# Supplementary material for: Hospital Security Searches Among Patients With Substance-Related Encounters
Source: JAMA Netw Open. 2025 Mar 18;8(3):e251068. doi: 10.1001/jamanetworkopen.2025.1068 (PMC11920837; doi:10.1001/jamanetworkopen.2025.1068)
Supplement: Supplement 2. — Data Sharing Statement [file jamanetwopen-e251068-s002.pdf]

Data Sharing Statement

Data
Data available: No

Additional Information
Explanation for why data not available: The data used in this study cannot be shared to protect the privacy of included patients and staff.
